# Utility of Three‐Dimensional Echocardiography for Ventricular Assessment in Congenital Heart Disease: A Comparative Retrospective Study

**DOI:** 10.1111/echo.70472

**Published:** 2026-05-12

**Authors:** Yoichiro Ishii, Takuya Fujisaki, Masayoshi Mori, Kumiyo Matsuo, Dai Asada, Tomomitsu Kanaya, Nanami Fujii, Sanae Tsumura, Hisaaki Aoki

**Affiliations:** ^1^ Department of Cardiology Osaka Women's and Children's Hospital Osaka Japan; ^2^ Department of Cardiovascular Surgery Osaka Women's and Children's Hospital Osaka Japan; ^3^ Department of Clinical Laboratory Osaka Women's and Children's Hospital Osaka Japan

**Keywords:** adult congenital heart disease, congenital heart disease, non‐invasive modality, three‐dimensional echocardiography, ventricular volume and functional evaluation

## Abstract

**Purpose:**

Accurate quantification of ventricular volumes and function is critical for managing congenital heart disease (CHD) and guiding surgical and interventional decisions. Although cardiac magnetic resonance imaging (CMR) and cardiac catheterization (CA) are gold standards, their use is limited by availability, invasiveness, and patient tolerance. Three‐dimensional echocardiography (3DE) offers a non‐invasive alternative; however, validation across modalities in heterogeneous CHD populations is limited. The aim of this study was to evaluate the clinical utility of 3DE in patients with CHD by comparison with against measurements obtained by CMR and CA.

**Methods:**

We retrospectively analyzed patients with CHD undergoing 3DE, CMR, and CA. Ventricular end‐diastolic volume (EDV), end‐systolic volume (ESV), ejection fraction (EF), and cardiac index (CI) were measured and compared across the three modalities. 3DE data were analyzed using Philips QLAB. Correlation coefficients, regression equations, and Bland–Altman analyses were used to assess agreement across modalities.

**Results:**

Thirty‐six patients were included between 2024 and 2025. Strong correlations were observed between 3DE and CMR (LVEDV *r* = 0.94, RVEDV *r* = 0.93) as well as 3DE and CA (LVEDV *r* = 0.86, RVEDV *r* = 0.89). 3DE systematically underestimated volumes (3DE < CMR < CA). EF showed excellent correlation across modalities, whereas CI exhibited greater variability. Bland–Altman analysis confirmed a systematic bias that remained within clinically acceptable limits for population‐level assessment, although individual‐based variability should be considered.

**Conclusions:**

3DE provides reliable volumetric and functional assessment in patients with CHD, with strong correlation with CMR and CA despite systematic underestimation of absolute values. 3DE is a practical noninvasive modality for serial follow‐up.

## Graphical abstract

Three‐dimensional echocardiography provides reliable ventricular assessment in patients with congenital heart disease, showing a strong correlation with cardiac magnetic resonance imaging and catheterization despite systematic underestimation.

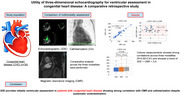

**Abbreviations**
2DE: two‐dimensional echocardiography
3DE: three‐dimensional echocardiography
CA: cardiac catheterization
CI: cardiac index
CO: cardiac output
CHD: congenital heart disease
CMR: cardiac magnetic resonance imaging
EDV: end‐diastolic volume
ESV: end‐systolic volume
LV: left ventricle
RV: right ventricle
SV: stroke volume.

## 1 INTRODUCTION

Accurate evaluation of ventricular volumes and function is essential in the management of patients with congenital heart disease (CHD), particularly for guiding therapeutic decisions, determining surgical indications, and monitoring long‐term outcomes [1, 2]. Ventricular function not only serves as a prognostic indicator but also directly influences the timing and selection of interventions. Traditionally, cardiac catheterization (CA) and cardiac magnetic resonance imaging (CMR) have been considered the reference standards for quantifying ventricular volumes and function in both pediatric and adult patients with CHD [3]. However, both modalities have inherent limitations. CA is invasive, and CMR, although highly accurate and reproducible, is costly, time‐consuming, and often requires sedation or general anesthesia in pediatric populations. In contrast, echocardiography remains the most widely available and non‐invasive modality for cardiac evaluation due to its accessibility, safety, and cost‐effectiveness [4]. Among echocardiographic techniques, three‐dimensional echocardiography (3DE) has gained increasing attention over the past 20 years as a valuable tool for assessing ventricular volumes and systolic function. Unlike conventional two‐dimensional echocardiography (2DE), which relies on geometric assumptions and planar views, 3DE offers volumetric analysis based on direct reconstruction of ventricular anatomy. This is particularly advantageous in CHD, where the complex and variable morphology of the ventricles often renders geometric assumptions inaccurate [5]. Recent advances in 3DE technology, including improvements in spatial and temporal resolution and the incorporation of semi‐automated analysis software, have further enhanced its clinical utility [6].

Previous studies have demonstrated that 3DE correlates well with CMR in assessing left ventricular (LV) and right ventricular (RV) volumes in patients with structurally normal hearts. For instance, Yanagi et al. reported a strong correlation between 3DE and CMR‐derived stroke volume (SV) and Qp/Qs ratios in patients with atrial septal defects using phase‐contrast CMR as the reference standard [7]. However, data remain limited regarding the comparative accuracy of 3DE in complex CHD, especially in children, where anatomical heterogeneity and acoustic window limitations pose additional challenges. Moreover, the validation of 3DE against both CMR and CA in a cohort that includes single ventricle physiology has not been sufficiently explored.

In patients with CHD, the accurate quantification of ventricular end‐diastolic volume (EDV), end‐systolic volume (ESV), and derived parameters such as SV, cardiac output (CO), and cardiac index (CI) is essential for optimal management. In single ventricle physiology, assessment of the systemic ventricle plays a pivotal role in determining surgical strategy, timing of staged palliation, and risk stratification [8].

In this context, the present study aimed to evaluate the clinical utility and accuracy of 3DE in a heterogeneous cohort of pediatric patients with CHD by comparing ventricular volumes and function obtained via 3DE with those obtained via CMR and CA. We hypothesized that 3DE‐derived measurements would show a strong correlation with those obtained from the established modalities, though with a tendency to underestimate absolute volume values. Additionally, we sought to assess the regression formulas between modalities and identify any systematic differences, particularly between systemic LV and RV measurements, to provide practical guidance for integrating 3DE into routine clinical decision‐making in CHD.

The significance of this investigation lies in its inclusion of both biventricular and single‐ventricle patients, as well as the simultaneous comparison of 3DE with both CMR and CA. Such triangulated validation is rarely reported in the literature and holds relevance for institutions where CMR or catheter‐based evaluations are not routinely accessible. Furthermore, as 3DE technology becomes increasingly available in pediatric cardiology units, establishing its reliability across a broad spectrum of CHD morphologies will be key to its widespread adoption.

Therefore, the primary objectives of this study were:

1. To evaluate the correlation between 3DE, CMR, and CA‐derived measurements of ventricular volumes and function in pediatric and young adult patients with CHD.
2. To determine systematic biases in 3DE measurements compared to CMR and CA, and to establish regression equations that can aid in clinical interpretation.
3. To assess whether 3DE provides sufficient accuracy for clinical decision‐making in both single‐ and biventricular repair patients.

By achieving these aims, we hope to promote the integration of 3DE as a non‐invasive, reliable, and accessible imaging modality in the comprehensive care of patients with CHD.

## 2 MATERIALS AND METHODS

### 2.1 Study Design and Population

This was a retrospective, observational, single‐center study conducted at the Osaka Women's and Children's Hospital, approved by the hospital's ethics committee (number 1743). Informed consent was obtained from all participants or their guardians in accordance with the Declaration of Helsinki. We enrolled consecutive patients with CHD who underwent 3DE, CMR, and CA for clinical evaluation between January 2024 and May 2025.

Inclusion criteria were:

1. Diagnosed CHD with planned CMR and CA for clinical indications.
2. Acquisition of technically adequate 3DE datasets within 2 months of CMR and CA.

Exclusion criteria included:

1. Poor echocardiographic acoustic windows precluding complete 3DE acquisition.
2. Contraindications to CMR (e.g., implanted ferromagnetic devices, severe claustrophobia, extensive metallic tattoos).
3. Significant arrhythmia during acquisition that affects data quality.

Patients were eligible for inclusion if 3DE, CMR, and CA studies were performed within 2 months, and if sufficient image quality allowed quantitative analysis of ventricular volumes and function. A total of 36 patients (median age 17 years, range 1–42 years; median weight 44.6 kg, range 9.1–67.7 kg) were included. Diagnoses included Tetralogy of Fallot (*n* = 8), single ventricle (*n* = 6), tricuspid atresia (*n* = 6), double outlet right ventricle (*n* = 3), transposition of the great arteries (*n* = 3), ventricular septal defect (*n* = 3), and other CHDs (*n* = 7). Eighteen patients had undergone biventricular repair, and the remaining 18 had single‐ventricle palliation.

### 2.2 Three‐Dimensional Echocardiography

All echocardiographic studies were performed using an EPIQ CVx ultrasound system (Philips Healthcare, Andover, MA, USA) equipped with X5‐1 matrix‐array transducers. Standard apical and subcostal windows were used according to the patient's age and habitus. For 3DE acquisitions, full‐volume datasets were obtained using a multi‐beat (4–6 beats) or single‐beat mode depending on rhythm stability and patient cooperation.

Analysis was performed using 3D Auto RV and Dynamic heart model software in EPIQ CVx 9.0.10 (Philips Ultrasound LCC, Netherlands). Endocardial borders of the systemic and subpulmonary ventricles were semi‐automatically detected and manually adjusted as necessary in end‐diastolic and end‐systolic frames. EDV, ESV, SV, CO, and CI were calculated. For single‐ventricle physiology, the systemic ventricle was analyzed using the same protocol.

### 2.3 Cardiac Magnetic Resonance

CMR was performed on a 1.5‐T scanner with GE Optima 450 W (GE HealthCare Technologies Inc., USA) with a phased‐array cardiac coil. Imaging included steady‐state free precession cine sequences in contiguous short‐axis slices covering the entire ventricle(s) from base to apex, as well as standard long‐axis views. Retrospective ECG‐gating was used. Ventricular volumes and function were analyzed using dedicated software (Ziostation 2, Amin Co., Japan) by experienced radiologists blinded to 3DE and CA results. Papillary muscles and trabeculations were included in the blood volume.

### 2.4 Cardiac Catheterization

CA was performed using the Alphenix INFX‐8000 V (Canon medical systems corporation, Japan), and these were under general anesthesia or sedation according to patient age and clinical status. Hemodynamic data, including pressures and oxygen saturations at multiple levels, were recorded. Ventricular volumes were calculated by angiography using the biplane area–length method. CI was calculated using the Fick principle.

### 2.5 Statistical Analysis

Continuous variables were expressed as mean ± standard deviation or median (interquartile range), depending on their distribution. Normality was tested using the Shapiro–Wilk test. Comparisons between modalities (3DE, CMR, CA) were performed using paired t‐tests or Wilcoxon signed‐rank tests. Correlations between methods were assessed by Spearman's correlation coefficient. Agreement was evaluated using Bland–Altman analysis, and regression equations were derived to assess systematic differences. Statistical significance was set as *p*‐value < 0.05. All statistical analyses were performed using EZR (Easy R), version 1.61 (Saitama Medical Center, Jichi Medical University, Saitama, Japan).

## 3 RESULTS

### 3.1 Patient Characteristics

A total of 36 patients with CHD who underwent 3DE, CMR, and CA within a 2‐month interval were included in the analysis. The cohort included 18 patients with biventricular physiology and 18 patients with single‐ventricle physiology. The median age was 17.0 years (range 1–42 years), and the median body weight was 44.6 kg (range 9.1–67.0 kg). The primary diagnoses included Tetralogy of Fallot (*n* = 8), tricuspid atresia (*n* = 6), single ventricle of undetermined morphology (*n* = 6), transposition of the great arteries (*n* = 3), double outlet right ventricle (*n* = 3), ventricular septal defect (*n* = 3), and other lesions (*n* = 7). Detailed baseline characteristics are summarized in Table 1.

**TABLE 1:** Patient characteristics.

Variable	*N* = 36
Age (years)	14.9 ± 8.8
Sex (M/F)	23/13
Height (cm)	139.1 ± 31.2
Weight (kg)	40.1 ± 19.7
Body surface area (m^2^)	1.23 ± 0.45
Univentricular/Biventricular cardiac disease	18(LV 12, RV 6)/18
Diagnosis of heart disease	
Tetralogy of Fallot	8
Tricuspid atresia	6
Single ventricle	6
Transposition of the great arteries	3
Double outlet right ventricle	3
Ventricular septal defect	3
Others	7
Extracardiac anomaly	
Right isomerism	4
Trisomy 21	2
22q11.2 deletion	1
Others	3

Abbreviation: LV, left ventricle; RV, right ventricle.

### 3.2 Correlation between 3DE and CMR

**FIGURE 1:**
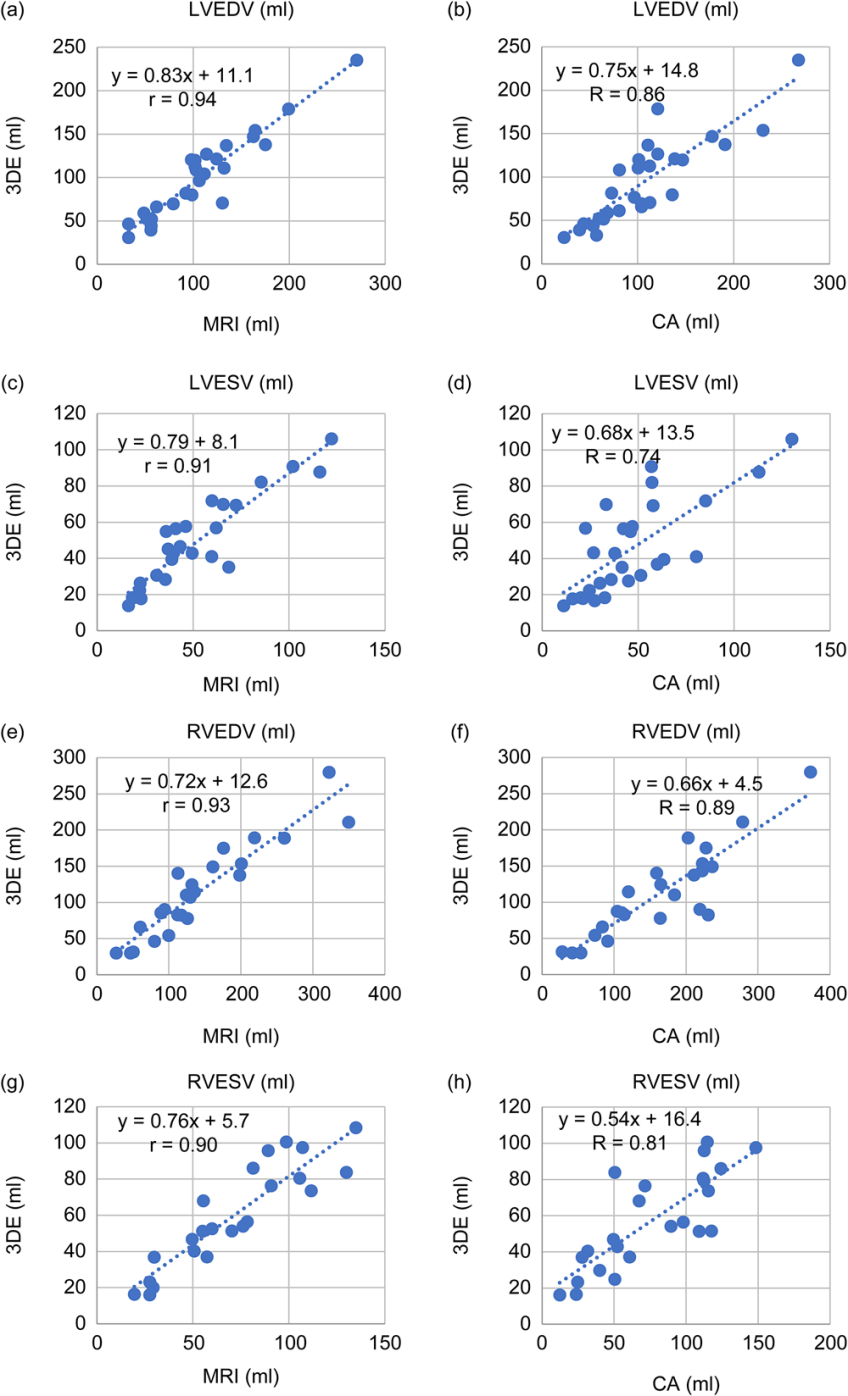
Correlation plots of 3DE versus CMR and CA for ventricular volumes. Scatter plots demonstrating correlations between 3DE and CMR (left column) and between 3DE and CA (right column) for LVEDV (a, b), LVESV (c, d), RVEDV (e, f), and RVESV (g, h). Regression lines and 95% confidence intervals are shown. Strong correlations were observed across all parameters, with correlation coefficients (*r*) highest for left ventricular measurements. 3DE: 3‐dimensional echocardiography, CA: catheter angiography, CMR: cardiac magnetic resonance imaging, LVEDV: left ventricular end‐diastolic volume, LVESV: left ventricular end‐systolic volume, RVEDV: right ventricular end‐diastolic volume, RVESV: right ventricular end‐systolic volume.

**FIGURE 2:**
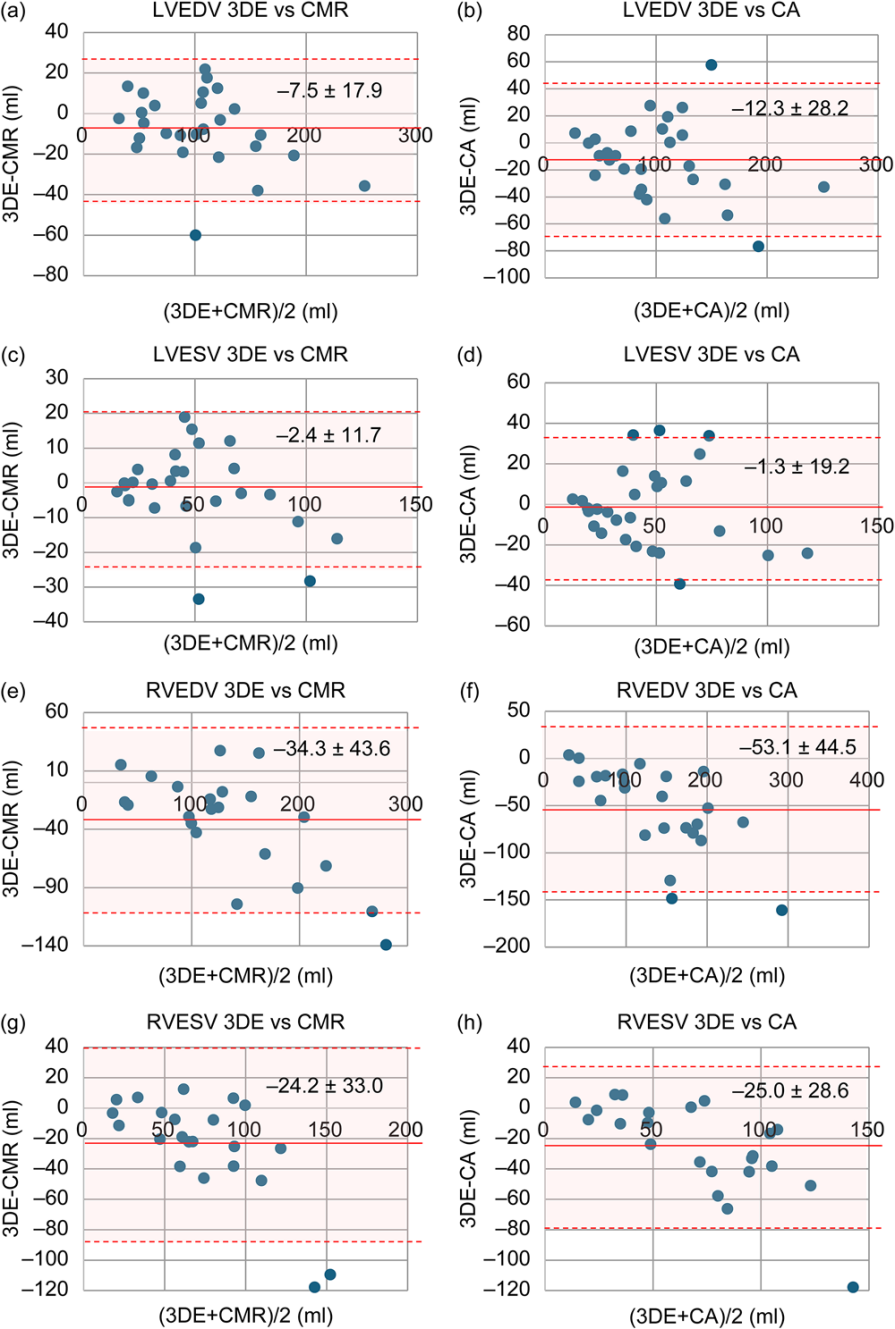
Bland–Altman analysis of 3DE versus CMR and CA for ventricular volumes. Bland–Altman plots comparing 3DE with CMR (left column) and 3DE with CA (right column) for LVEDV (a, b), LVESV (c, d), RVEDV (e, f), and RVESV (g, h). Mean bias (solid line) and limits of agreement (± 1.96 SD; dashed lines) are displayed. 3DE systematically underestimated volumes relative to both modalities, with the smallest bias for left ventricular volumes and largest for right ventricular volumes. 3DE: 3‐dimensional echocardiography, CA: catheter angiography, CMR: cardiac magnetic resonance imaging, LVEDV: left ventricular end‐diastolic volume, LVESV: left ventricular end‐systolic volume, RVEDV: right ventricular end‐diastolic volume, RVESV: right ventricular end‐systolic volume.

3DE demonstrated strong correlations with CMR for LVEDV (*r* = 0.94, *p* < 0.001; regression: 3DE = 0.89 × CMR + 11.1) and LVESV (*r* = 0.91, *p* < 0.001; regression: 3DE = 0.79 × CMR + 8.1; Figure 1a,c). Bland–Altman analysis confirmed systematic underestimation by 3DE, with a mean bias of –7.5 ± 17.9 mL for LVEDV (LoA: –42.6 to 27.7 mL) and –2.4 ± 11.7 mL for LVESV (LoA: –25.3 to 20.5 mL; Figure 2a,c).

For RV volumes, correlation was strong for RVEDV (r = 0.93, p < 0.001; regression: 3DE = 0.72 × CMR + 12.6) and moderate for RVESV (r = 0.90, p < 0.001; regression: 3DE = 0.76 × CMR + 5.7; Figure 1e,g). Bias was more pronounced than in LV measurements (RVEDV bias: –35.3 ± 43.6 mL, LoA: –119.7 to 51.1 mL; RVESV bias: –24.2 ± 33.0 mL, LoA: –88.9 to 40.6 mL; Figure 2e,g).

**FIGURE 3:**
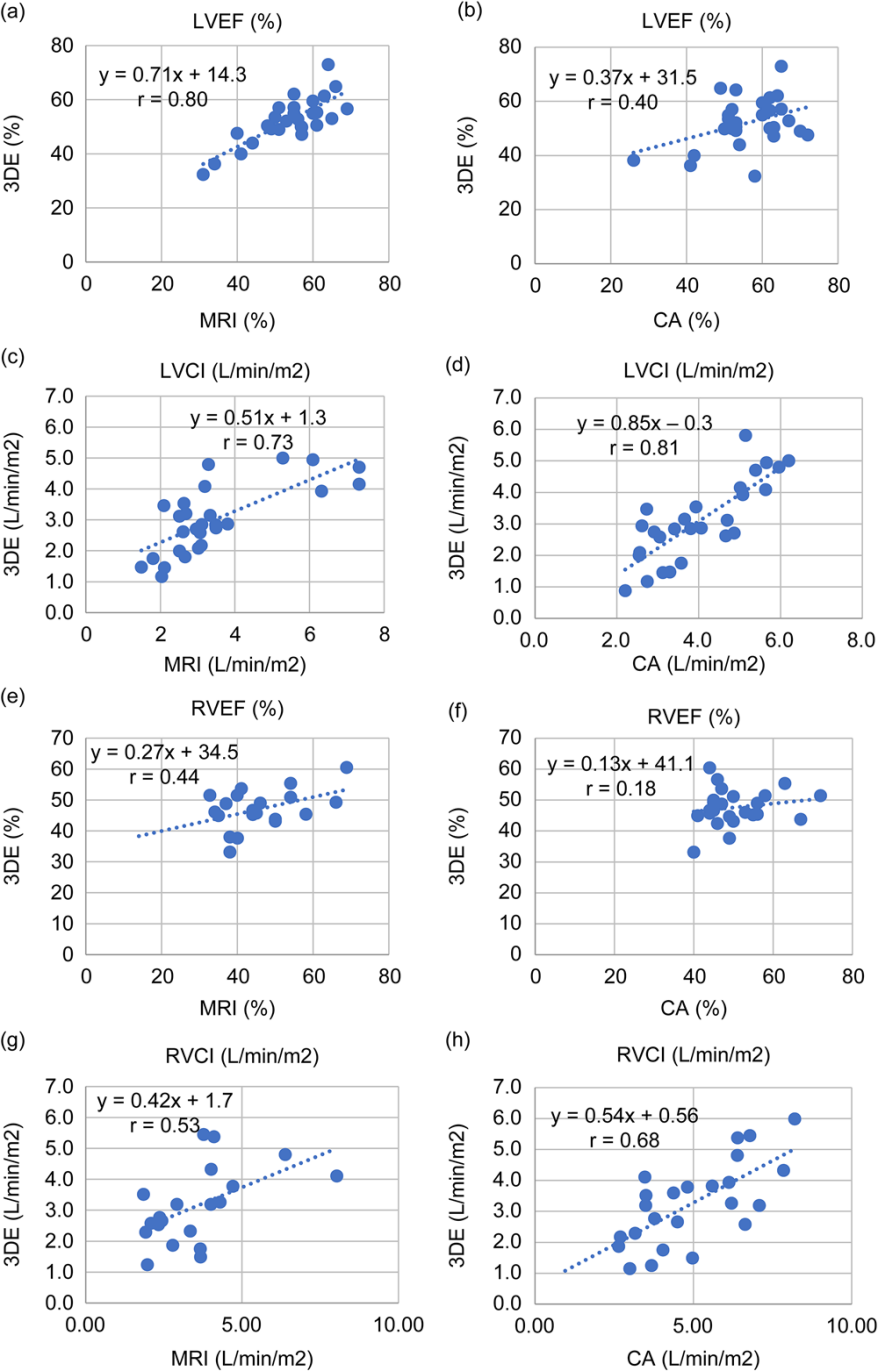
Correlation plots of 3DE versus CMR and CA for ejection fraction and cardiac index. Scatter plots showing correlations of EF and CI between 3DE and CMR (left panels) and between 3DE and CA (right panels). EF (a, b, e, f) demonstrated strong correlation across modalities, whereas CI (c, d, g, h) showed greater variability, reflecting compounding errors in stroke volume estimation and body surface area normalization. 3DE: 3‐dimensional echocardiography, CA: catheter angiography, CI: cardiac index, CMR: cardiac magnetic resonance imaging, EF: ejection fraction.

For functional parameters, LVEF exhibited strong correlation (*r* = 0.80, *p* < 0.001; regression: 3DE = 0.71 × CMR + 14.3), whereas RVEF correlation was weaker (*r* = 0.44, *p* = 0.246; regression: 3DE = 0.27 × CMR + 34.5; Figure 3a,e).

Cardiac index for the LV (LVCI) showed strong correlation (*r* = 0.73, *p* < 0.001; regression: 3DE = 0.51 × CMR + 1.3), while RVCI correlation was moderate (*r* = 0.53, *p* < 0.001; regression: 3DE = 0.42 × CMR + 1.7; Figure 3c,g).

**FIGURE 4:**
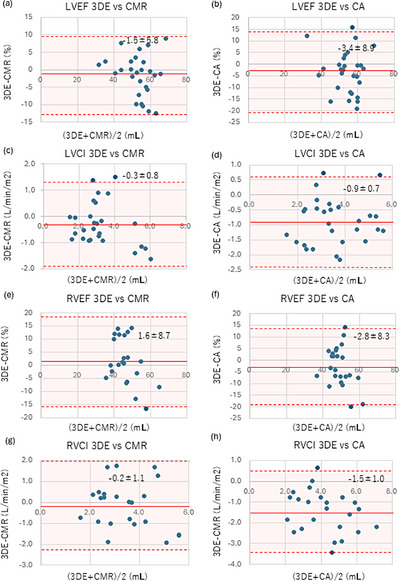
Bland–Altman analysis of 3DE versus CMR and CA for ventricular function. Bland–Altman plots comparing 3DE with CMR (left column) and 3DE with CA (right column) for LVEF (a, b), LVCI (c, d), RVEF (e, f), and RVCI (g, h). The mean bias (solid line) and limits of agreement (±1.96 SD; dashed lines with hatched area) are shown. 3DE demonstrated good agreement for LVEF and RVEF among both modalities, whereas LVCI and RVCI were slightly underestimated.

Bland–Altman analysis showed relatively small biases for LVEF and RVEF compared with that of CMR (LVEF bias: −1.5 ± 5.8%, LoA: −13.0 to 9.9%; RVEF bias: 1.6 ± 8.7%, LoA: −15.5 to 18.7%; Figure 4a,e), indicating acceptable agreement at the population level. Similarly, LVCI and RVCI showed relatively small biases (LVCI bias: −0.3 ± 0.8 L/min/m^2^, LoA: −1.9 to 1.3 L/min/m^2^; RVCI bias: −0.2 ± 1.1 L/min/m^2^, LoA: −2.3 to 2.0 L/min/m^2^; Figure 4c,g).

### 3.3 Correlation between 3DE and CA

When compared with CA, LVEDV correlation remained strong (*r* = 0.86, *p* < 0.001; regression: 3DE = 0.75 × CA + 14.8) and LVESV (*r* = 0.74, *p* < 0.001; regression: 3DE = 0.68 × CA + 13.5; Figure 1b,d).

Bland–Altman analysis confirmed systematic underestimation by 3DE, with a mean bias of –12.3 ± 28.2 mL for LVEDV (LoA: –67.6 to 42.9 mL) and –1.3 ± 19.2 mL for LVESV (LoA: –38.9 to 36.2 mL; Figure 2b,d).

RVEDV correlation was strong (*r* = 0.89, *p* < 0.001; regression: 3DE = 0.66 × CA + 4.5) with the largest bias in Bland–Altman analysis observed among all measurements (–53.1 ± 44.5 mL, LoA: –140.4 to 34.1 mL; Figures 1f and 2f). RVESV correlation was also strong (*r* = 0.81, *p* < 0.001; regression: 3DE = 0.54 × CA + 16.4) with a bias in Bland–Altman analysis of –25.0 ± 28.6 mL (LoA: –81.1 to 31.1 mL; Figures 1h and 2h).

For functional parameters, LVEF showed moderate correlation (*r* = 0.40, *p* = 0.51), whereas RVEF correlation was weaker (*r* = 0.18, *p* = 0.56; Figure 3b,f).

LVCI correlation was strong (*r* = 0.81, *p* < 0.001; regression: 3DE = 0.81 × CA + 0.3), whereas RVCI correlation was also strong (*r* = 0.68, *p* < 0.001; regression: 3DE = 0.54 × CA + 0.56; Figure 3d,h).

Bland–Altman analysis showed relatively small biases for LVEF and RVEF compared with that of CA (LVEF bias: −3.4 ± 8.9%, LoA: −20.8 to 14.1%; RVEF bias: −2.8 ± 8.3%, LoA: −19.1 to 13.6%; Figure 4b,f), indicating acceptable agreement at the population level, although individual‐based variability should be considered. Similarly, LVCI and RVCI showed relatively small biases (LVCI bias: −0.9 ± 0.7 L/min/m^2^, LoA: −2.4 to 0.6 L/min/m^2^; RVCI bias: −1.5 ± 1.0 L/min/m^2^, LoA: −3.4 to 0.5 L/min/m^2^; Figure 4d,h). However, consistent with the comparison to CMR, ventricular volume measurements demonstrated wider limits of agreement, particularly for RV volumes, and should, therefore, be interpreted with caution at the individual level.

### 3.4 Systematic Trends across Modalities

**FIGURE 5:**
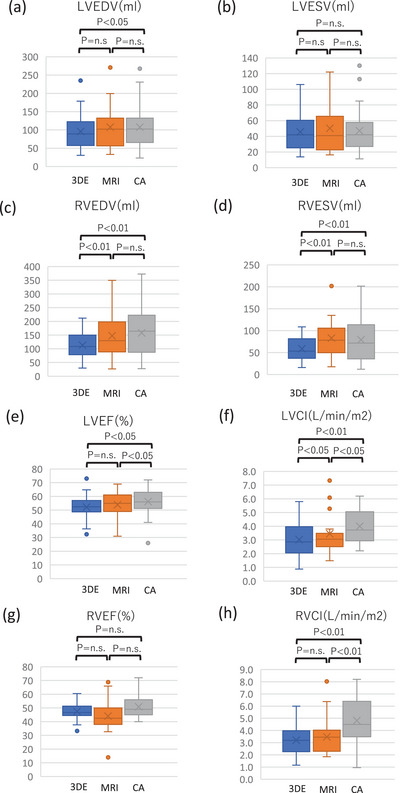
Box‐and‐whisker plots comparing ventricular volumes, EF, and CI across modalities. Box‐and‐whisker plots depicting measurements from 3DE, CMR, and CA for LVEDV (a, c), LVESV (b, d), RVEDV, RVESV, EF (e, g), and CI (f, h). Boxes represent IQR, horizontal lines indicate medians, whiskers represent maximum and minimum values, and dots represent individual patient data. Volumes showed a consistent trend of 3DE < CMR < CA. EF values were comparable across modalities, whereas CI values showed modest differences. 3DE: 3‐dimensional echocardiography, CA: catheter angiography, CI: cardiac index, CMR: cardiac magnetic resonance imaging, EF: ejection fraction, IQR: interquartile range, LVEDV: left ventricular end‐diastolic volume, LVESV: left ventricular end‐systolic volume, RVEDV: right ventricular end‐diastolic volume, RVESV: right ventricular end‐systolic volume.

Box‐and‐whisker plots (Figure 5) demonstrate the consistent trend of 3DE < CMR < CA for all ventricular volumes. Overall, 3DE demonstrated a strong correlation with both CMR and CA for ventricular volumes and EF. Systematic underestimation of volumes was observed, particularly for RV measurements, with the bias being smaller for CMR than CA. EF values were consistent across modalities, while CI showed greater variability.

## 4 DISCUSSION

Accurate quantification of ventricular volumes and function is essential for managing CHD, guiding surgical timing, and predicting outcomes [1, 2]. CMR is considered the reference standard for noninvasive volumetric assessment due to its reproducibility and freedom from acoustic window limitations [9, 10]. CA remains important for hemodynamic assessment, but is invasive and carries procedural risk [11]. Both modalities are limited by availability, patient tolerance, and, in pediatrics, the need for anesthesia.

3DE offers a non‐invasive, widely available alternative, overcoming the geometric assumptions of 2DE by reconstructing true ventricular geometry [12]. Technological advances have improved its spatial/temporal resolution and automated border detection, making it increasingly viable even in complex CHD. However, direct validation of 3DE against both CMR and CA in heterogeneous CHD cohorts remains limited.

### 4.1 Principal Findings

The present study is, to the best of our knowledge, one of the few to evaluate 3DE against both CMR and CA in the same cohort of pediatric and young adult patients with CHD. The main findings can be summarized as follows:

1. Strong correlations were observed between 3DE and CMR, as well as between 3DE and CA, for most volumetric and functional parameters. A systematic bias was evident between each modality, establishing a volumetric hierarchy of 3DE < CMR < CA. Based on these data, we estimated that CMR volume is approximately 1.2× of 3DE volume, and CA volume is approximately 1.4× of 3DE volume.
2. The underestimation by 3DE was more pronounced for RV volumes compared with that of LV. Bland–Altman analyses confirmed these systematic biases but suggested that the limits of agreement were within clinically acceptable ranges for volumetric parameters; however, the limits were relatively wide, particularly for RVEDV.
3. Ejection fraction and CI demonstrated moderate agreement across modalities, which showed greater variability, especially in RV‐derived indices.

### 4.2 Comparison With Previous Studies

Our finding of strong correlations between 3DE and CMR, especially for LV volumes, aligns with earlier reports in both normal and abnormal hearts [13, 14]. In a study by Wu, 3DE underestimated LVEDV by approximately 10%–15% compared with that of CMR, which is consistent with the magnitude of bias observed in our data. The systematic underestimation is likely multifactorial: suboptimal endocardial border delineation due to limited spatial resolution, particularly in apical regions; difficulty in imaging basal segments; and residual errors in semi‐automated contour detection.

Compared with LV, for RV volumes, the larger biases and weaker correlations were previously reported [15, 16]. The RV's complex crescentic geometry, heavy trabeculation, and anterior position within the thorax limit echocardiographic visualization, especially in patients with post‐surgical changes [17]. Our observation that RVEDV and RVESV biases were substantially larger than those for LV volumes underscores this challenge and suggests that 3DE‐derived RV volumes should be interpreted with caution, particularly when used to guide the timing of interventions, such as pulmonary valve replacement.

The stepwise volumetric hierarchy observed (3DE < CMR < CA) mirrors findings from studies comparing CA‐derived angiographic volumes with CMR. CA tends to yield larger volumes, partly due to differences in imaging conditions (general anesthesia, low afterload from sedation) and the use of contrast ventriculography, which may overestimate cavity size by including trabecular recesses. CMR, although less prone to such overestimation, can still differ from true physiologic volumes due to slice thickness, breath‐hold technique, and variability in contouring protocols [18].

3DE's strong correlation for LV volumes and EF supports its role in routine CHD follow‐up, especially for detecting longitudinal trends. This could reduce dependence on CMR and CA, minimizing patient burden and cost. However, baseline calibration against CMR is recommended when possible, especially for RV volumes and functional parameters. For conditions where RV size/function dictates intervention (e.g., repaired tetralogy of Fallot), CMR is the preferred modality, and 3DE might be used to guide interventional decisions. CI variability, especially that derived from the RV morphology, can limit accurate hemodynamic quantification—especially relevant in single‐ventricle physiology where systemic output assessment is critical.

The strengths of this study include head‐to‐head comparison of 3DE with both CMR and CA within a short temporal window, minimizing the impact of physiologic changes between measurements. The inclusion of both biventricular and single‐ventricle anatomies enhances the generalizability of our findings across the CHD spectrum. Furthermore, by providing regression equations between modalities, our results offer practical tools for clinicians to approximate reference‐standard values from 3DE measurements.

### 4.3 Limitations

Several limitations must be acknowledged. First, the sample size was relatively small, reflecting the single‐center design, short study period, and the need for all three imaging modalities within a limited time frame. Second, our study excluded patients with inadequate echocardiographic windows, which may bias results toward those with more favorable imaging conditions. Finally, all echocardiographic analyses were performed by experienced operators in a tertiary center; results may not be reproducible in less specialized settings.

## 5 Conclusion

This study demonstrates that 3DE provides reliable volumetric and functional assessment in CHD, with strong correlations to CMR and CA despite a consistent tendency to underestimate absolute volumes. The bias is systematic, most pronounced for RV volumes, but remains within clinically acceptable limits for follow‐up. Integrating 3DE into routine CHD surveillance may reduce reliance on invasive or resource‐intensive modalities, improving patient comfort and resource utilization. CMR should be reserved for baseline calibration and at key clinical milestones, particularly for systemic RV or complex single‐ventricle anatomies.

## Funding

This research did not receive any specific grant from funding agencies in the public, commercial, or not‐for‐profit sectors.

## Ethics Statement

The Osaka Women's and Children's Hospital Ethics Committee (number 1743) granted ethics approval.

## Consent

Informed consent was obtained from all participants or their guardians in accordance with the Declaration of Helsinki.

## Conflicts of Interest

The authors declare no conflicts of interest.

## Data Availability

All data generated or analyzed during this study are included in this article. Further inquiries can be directed to the corresponding author.
